# Supplementary material for: Modeling glioblastoma heterogeneity as a dynamic network of cell states
Source: Mol Syst Biol. 2021 Sep 16;17(9):e10105. doi: 10.15252/msb.202010105 (PMC8444284; doi:10.15252/msb.202010105)
Supplement: Supplementary file 6 — Source Data for Figure 5 [file MSB-17-e10105-s004.zip › Figure5A_sourcedata/GSEA_3017/nosigdb_stateA.GseaPreranked.1622795026485/NB_KI_CLUSTER18.html]

Details for gene set NB\_KI\_CLUSTER18[GSEA]

|  || Dataset | state53017 |
| Phenotype | NoPhenotypeAvailable |
| Upregulated in class | na\_neg |
| GeneSet | NB\_KI\_CLUSTER18 |
| Enrichment Score (ES) | -0.6875536 |
| Normalized Enrichment Score (NES) | -3.0764878 |
| Nominal p-value | 0.0 |
| FDR q-value | 0.0 |
| FWER p-Value | 0.0 |
Table: GSEA Results Summary

  

Fig 1: Enrichment plot: NB\_KI\_CLUSTER18      
 Profile of the Running ES Score & Positions of GeneSet Members on the Rank Ordered List

  

| PROBE | GENE SYMBOL | GENE\_TITLE | RANK IN GENE LIST | RANK METRIC SCORE | RUNNING ES | CORE ENRICHMENT || 1 | VIM |  |  | 46 | 0.543 | -0.0218 | No |
| 2 | ITGB1 |  |  | 256 | 0.346 | -0.2229 | No |
| 3 | NR2F2 |  |  | 303 | 0.324 | -0.2552 | No |
| 4 | MT2A |  |  | 473 | 0.275 | -0.4181 | No |
| 5 | ZFP36L1 |  |  | 532 | 0.264 | -0.4658 | No |
| 6 | CD99 |  |  | 604 | -0.252 | -0.5277 | No |
| 7 | RGS16 |  |  | 620 | -0.260 | -0.5308 | No |
| 8 | SOCS3 |  |  | 651 | -0.270 | -0.5491 | No |
| 9 | THY1 |  |  | 663 | -0.277 | -0.5472 | No |
| 10 | IGFBP7 |  |  | 780 | -0.368 | -0.6504 | No |
| 11 | SPARC |  |  | 782 | -0.369 | -0.6337 | No |
| 12 | LHFPL6 |  |  | 796 | -0.381 | -0.6289 | No |
| 13 | FILIP1L |  |  | 835 | -0.428 | -0.6480 | No |
| 14 | PRSS23 |  |  | 874 | -0.486 | -0.6642 | Yes |
| 15 | CALD1 |  |  | 889 | -0.527 | -0.6535 | Yes |
| 16 | A2M |  |  | 898 | -0.545 | -0.6356 | Yes |
| 17 | CYTOR |  |  | 918 | -0.591 | -0.6270 | Yes |
| 18 | CST3 |  |  | 933 | -0.680 | -0.6089 | Yes |
| 19 | CEBPD |  |  | 939 | -0.690 | -0.5810 | Yes |
| 20 | IFITM3 |  |  | 954 | -0.830 | -0.5557 | Yes |
| 21 | CDKN1A |  |  | 969 | -0.987 | -0.5229 | Yes |
| 22 | LGALS3 |  |  | 970 | -0.989 | -0.4754 | Yes |
| 23 | CYR61 |  |  | 978 | -1.296 | -0.4204 | Yes |
| 24 | ID3 |  |  | 981 | -1.505 | -0.3502 | Yes |
| 25 | IGFBP5 |  |  | 983 | -1.595 | -0.2746 | Yes |
| 26 | SPARCL1 |  |  | 984 | -1.810 | -0.1877 | Yes |
| 27 | CCL2 |  |  | 985 | -1.881 | -0.0974 | Yes |
| 28 | NEAT1 |  |  | 986 | -2.049 | 0.0010 | Yes |
Table: GSEA details [plain text format]

  

Fig 2: NB\_KI\_CLUSTER18: Random ES distribution      
 Gene set null distribution of ES for **NB\_KI\_CLUSTER18**
